# Factors Associated With Craniocervical and Otological Symptoms in Healthcare Workers During Covid‐19 Pandemic: A Cross‐Sectional Study

**DOI:** 10.1111/joor.70099

**Published:** 2025-11-10

**Authors:** Anita Almeida Gonzaga, Jade Louise Alves Macedo Padilha Silva, Rafaella Silva dos Santos Aguiar Gonçalves, Luiz Felipe Tavares, Álvaro Campos Cavalcanti Maciel, Karyna Myrelly Oliveira Bezerra Figueiredo Ribeiro

**Affiliations:** ^1^ Department of Physical Therapy Federal University of Rio Grande do Norte Natal Brazil; ^2^ Postgraduate Program in Physical Therapy (PPGFt), Department of Physical Therapy Federal University of São Carlos São Carlos Brazil; ^3^ Postgraduate Program in Physical Therapy (PPGFST) Federal University of Rio Grande do Norte Natal Brazil

**Keywords:** headache, health personnel, personal protective equipment, temporomandibular joint disorders, tinnitus

## Abstract

**Background:**

Increased personal protective equipment (PPE) use, working hours and psychological disorders were observed among healthcare workers during the COVID‐19 pandemic, possibly leading to craniocervical and otological symptoms.

**Objective:**

This study aimed to identify factors associated with craniocervical and otological symptoms in healthcare workers during the COVID‐19 pandemic.

**Methods:**

A total of 147 healthcare workers in the care of patients with COVID‐19 were included. Craniocervical symptoms (headache, orofacial pain and neck pain), otological symptoms and PPE use before and during the pandemic were assessed. Pearson's chi‐square, McNemar's tests, the paired t‐test and a binary logistic regression were used for analysis.

**Results:**

The onset of symptoms during the pandemic was 44.2% for headache, 36.7% for orofacial pain, 38.0% for neck pain and 54.4% for otological symptoms. Worsening of pre‐existing symptoms was observed for headache (59.5%), orofacial pain (60.8%), neck pain (38.7%), and otological symptoms (29.4%). Depressive symptoms, associated symptoms and co‐occurrence of symptoms during the pandemic were associated with the onset of craniocervical symptoms (OR 2.42–3.99). The use and duration of surgical masks before the pandemic and face shield and orofacial pain during the pandemic were associated factors to the onset of otological symptoms (OR 2.98–3.89). Working in the nursing ward, female sex, lack of physical activity and previous medical history were associated with the worsening of pre‐existing craniocervical symptoms (OR 3.25–4.72).

**Conclusion:**

Craniocervical and otological symptoms increased among healthcare workers during the COVID‐19 pandemic. Addressing biopsychosocial factors may mitigate the health burden and improve the quality of care.

## Graphical abstract

Healthcare workers experienced increased craniocervical and otological symptoms during the COVID‐19 pandemic.

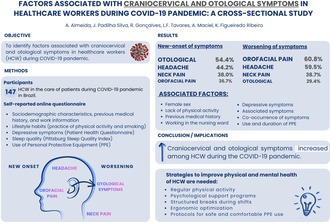

## 1 Introduction

The coronavirus disease 2019 (COVID‐19), initially detected in Wuhan, China, quickly escalated into a global pandemic, as declared by the World Health Organization (WHO) [1]. To reduce transmission, the use of personal protective equipment (PPE), particularly face masks, became essential [2], especially for healthcare workers who faced high exposure to the severe acute respiratory syndrome coronavirus type 2 (SARS‐CoV‐2).

However, prolonged PPE was associated with adverse physical and psychological effects, including ‘de novo’ headaches, fatigue and increased stress [3, 4]. These factors, combined with workload and the fear of contaminating themselves and family members, contributed to high rates of anxiety, exhaustion, insomnia and depression among healthcare workers, ultimately impairing their health, performance and quality of life [5, 6, 7, 8, 9]. Notably, such cumulative physical and psychological burdens may play a significant role in the manifestation of painful processes [10, 11].

During the COVID‐19 pandemic, headache was reported as one of the most prevalent complaints among healthcare workers [4, 12]. PPE‐related compression of trigeminal and occipital nerves may activate the trigeminocervical complex, a key mechanism in headache and orofacial pain [13]. Additionally, neck pain was also reported as common among healthcare workers during the COVID‐19 pandemic [14], and likely influenced by muscle tension, stress and workload [15]. Importantly, individuals with concomitant headache and orofacial symptoms have more disability compared to individuals with only orofacial complaints [16]. Temporomandibular disorders (TMD), frequently associated with psychological and otological symptoms (e.g., tinnitus, dizziness, ear fullness), exemplify the complex interplay of anatomical, neurological and psychosocial factors [17, 18, 19]. Therefore, understanding the functional dynamics of the trigeminocervical complex is essential in the pathophysiology of craniocervical pain [20].

Given the anatomical and functional interconnection of craniocervical structures, the pandemic context may have contributed to the onset or worsening of headache, neck pain, orofacial and otological symptoms in healthcare workers, which may negatively impact work and quality of life. Although headache has been widely reported as aggravated during the pandemic, the relationship between the other craniocervical complaints and factors such as PPE use, workload, lifestyle and psychological distress needs further investigation.

Therefore, this study aimed to identify factors associated with craniocervical and otological symptoms in healthcare workers during COVID‐19. Secondarily, the objectives were to determine the prevalence of these symptoms before and during the pandemic, to investigate factors associated with the worsening of pre‐existing craniocervical symptoms and to identify factors associated with the new onset of craniocervical complaints.

## 2 Methods

### 2.1 Study Design

This cross‐sectional study was conducted between September 2020 and April 2022 at the Department of Physical Therapy of the Federal University of Rio Grande do Norte (UFRN). This study followed the STROBE guidelines [21]. This study was approved by the local research ethics committee of the same institution (number 36193520.9.0000.5537). All research procedures were in accordance with the resolution 466/12 of the National Health Council and the Helsinki Declaration. Individuals' data stored in the virtual document are confidential and stored in Google Drive, which can only be accessed by the responsible researchers. A written informed consent was obtained from each participant.

### 2.2 Population and Sampling

The study population was healthcare workers, aged 18 years and older, who worked in the care of patients infected or suspected of COVID‐19 (with no restriction of department or level of care), from any region of Brazil. Individuals willing to participate answered a self‐applied online questionnaire (Google Forms platform), which contained guidelines, an informed consent form, and the questionnaires. Individuals who did not complete the questionnaire were excluded. Individuals were recruited through social media dissemination (Facebook, Instagram and WhatsApp), e‐mail and through the online system of the university (XXX).

### 2.3 Assessment Procedures

All individuals completed an online self‐administered questionnaire adapted from Ong et al. (2020) [12], through the Google Forms platform. The questionnaire contained 10 main components, namely:

1. Sociodemographic characteristics: identification; age; sex; profession; region; height, weight and body mass index (BMI).
2. Previous medical history: previous history of cardiovascular, respiratory, orthopaedic, rheumatologic, hormonal, metabolic, immunologic and neuropsychic disorders/diseases; diabetes mellitus; use of antidiabetic, contraceptive, anxiolytic, antidepressant, antihypertensive and anti‐inflammatory medications.
3. Work information: department, working hours before and during the pandemic; absences from work due to COVID‐19.
4. Lifestyle habits: self‐reported practice of physical activity (yes or no) and smoking (yes, used to or never).
5. Sleep quality: assessed by the Pittsburgh Sleep Quality Index (PSQI‐BR). The questionnaire assesses seven domains: subjective sleep quality, sleep latency, sleep duration, sleep efficiency, sleep disturbances, medication use and daily dysfunction. Each component encompasses different questions and has a specific calculation to assign a score from zero to three points, with an overall score of up to 21 points. Score above five indicate poor sleep quality of the individual. It is a valid and reliable instrument for this assessment, being easily understood and answered [22].
6. Depressive symptoms: screened through the Patient Health Questionnaire—two items (PHQ‐2) [23]. The PHQ‐2 assesses the frequency of depressive symptoms and anhedonia and is a valid instrument for the Brazilian population. The items are scored on a Likert scale from zero to three, and the total score can reach up to six points. Total scores equal to or greater than three points indicate probable depression.
7. Use of PPE: type (surgical masks, N95/FFP2 masks, goggles, face shields, caps), average number of days of use in the last 30 days; average daily hours of use.
8. Craniocervical and otological symptoms: participants were asked about the symptoms before and during the COVID‐19 pandemic, including orofacial pain (pain in the face or temporomandibular region), headache (pain in the cephalic segment/headache), and neck pain (pain in the cervical region). Otological symptoms considered the self‐reported of tinnitus (perception of sound in the absence of external sound), ear fullness (sensation of a full ear or pressure in the ear), hearing loss, dizziness (spatial disorientation), and vertigo (rotating sensation). For positive responses, symptoms intensity was assessed using the 11‐point Numerical Rating Scale (NRS 0–10) [24, 25]. The onset of symptoms was considered when symptoms were absent before the pandemic and present during the pandemic. The worsening of pre‐existing symptoms was considered based on the increase in symptom intensity (NRS) during the pandemic compared to before the pandemic. The number of worsening cases was also assessed (i.e., symptoms that were present before the pandemic and increased intensity during pandemic). The rate of increase considered the number of individuals with pre‐existing symptoms and those with new symptoms during the pandemic, expressed as a percentage. The average number of days per month that healthcare workers reported experiencing symptoms was also assessed.
9. Associated symptoms: other musculoskeletal pain complaints, nausea, vomiting, fatigue and anxiety before or during the pandemic.

### 2.4 Statistical Analysis

All analyses were performed using the statistical program SPSS version 20.0. The Kolmogorov–Smirnov test was used to verify the normality distribution of the data. Then, descriptive statistics were performed to characterise the sample and the results were presented as mean and standard deviation for parametric data. Categorical data were expressed as absolute and relative frequencies. Pearson's chi‐square and McNemar's tests were used to test associations, the Student's t‐test to compare onset and worsening of pre‐existing symptoms regarding each independent variable, and the paired t‐test was used to compare before and during the COVID‐19 pandemic.

Finally, to identify the factors associated with symptoms in healthcare workers a binary logistic regression was performed, using the backward method (Wald) and adjusted for age and sex. A *p* value ≤ 0.10 was first considered to include the potential variables associated with the onset and worsening of pre‐existing symptoms. The associations in which one of the cells had less than five observations were not included in the model, as they could interfere with the results and increase the confidence intervals. A 95% confidence interval and a significance level of *p* < 0.05 were adopted for all tests.

## 3 Results

A total of 168 individuals received the questionnaires, from which 157 agreed to participate and 147 completed all questionnaires. Table 1 describes sample characteristics. The mean age was 35 ± 9.7 years. Most individuals were women (79.6%), physiotherapists (43.5%), worked and lived in the northeast region of Brazil (83.7%), were overweight or obese (52.4%), worked in the intensive care unit (43.5%), and had a weekly workload greater than 40 h (25.2%). Most healthcare workers had poor sleep quality (63.9%) and almost a third had probable depression (29.9%).

**TABLE 1:** Sample characteristics (*n* = 147).

Variable	*n* (%)	Mean ± SD
Age (years)		35 ± 9.7
Sex		
Female	117 (79.6%)	
Male	30 (20.4%)	
Profession		
Physiotherapist	64 (43.5%)	
Physician	26 (17.7%)	
Nurse	24 (16.3%)	
Nursing technician	7 (4.8%)	
Others	26 (17.7%)	
Region		
Northeast	123 (83.7%)	
Southeast	19 (12.9%)	
North	02 (1.4%)	
South	02 (1.4%)	
Midwest	01 (0.7%)	
BMI		
Average weight	68 (46.3%)	
Overweight and obese	77 (52.4%)	
Underweight	2 (1.4%)	
Previous medical history		
No	77 (52.4%)	
Yes	70 (47.6%)	
Medication use		
No	85 (57.8%)	
Yes	62 (42.2%)	
Institution		
Public	99 (67.3%)	
Private	19 (12.9%)	
Public and private	29 (19.7%)	
Department^a^		
Urgency and emergency	40 (27.2%)	
ICU	64 (43.5%)	
Nursing ward	39 (26.5%)	
Outpatient	24 (16.3%)	
Primary care	30 (20.4%)	
Weekly workload		
≤ 40 h	110 (74.8%)	
> 40 h	37 (25.2%)	
Sick leave due to COVID		
No	96 (65.3%)	
Yes	43 (29.2%)	
Unsure	8 (5.5%)	
Smoking		
Yes	05 (3.4%)	
Used to	06 (4.1%)	
Never	136 (92.5%)	
Sleep quality		
Good	53 (36.1%)	
Bad	94 (63.9%)	
Depressive symptoms		
Unlikely depression	103 (70.1%)	
Probable depression	44 (29.9%)	

Abbreviations: BMI, body mass index; ICU, intensive care unit; SD, standard deviation.

^a^ Some individuals worked in more than one department.

Different patterns of PPE use by healthcare workers were observed before and during the pandemic, according to working department. The surgical mask was the most used PPE before the pandemic (73.5%) and the N95/FFP2 mask (90.5%) during the pandemic. An increase in the days per month using PPE was observed (*p* < 0.001) and an increase in hours/day for all PPE. Practicing physical activity before the pandemic was higher compared to during (*p* < 0.001). In addition, an increase of associated symptoms (nausea and vomiting, fatigue and anxiety) was also observed (*p* < 0.001). Table 2 summarises these results.

**TABLE 2:** Comparison of the prevalence and hours of PPE use, physical activity practice and associated symptoms of healthcare workers before and during the COVID pandemic (*n* = 147).

Outcomes	Before pandemic *n* (%) or mean ± SD	During pandemic *n* (%) or mean ± SD	*p*
PPE usage			
Surgical masks (*n* = 147)			
Yes	108 (73.5%)	105 (71.4%)	0.798
No	39 (26.5%)	42 (28.6%)	
N95/FFP2 (*n* = 147)			
Yes	30 (20.4%)	133 (90.5%)	**0.001** *
No	117 (79.6%)	14 (9.5%)	
Face shield (*n* = 147)			
Yes	2 (1.4%)	113 (76.9%)	**0.001** *
No	145 (98.6%)	34 (23.1%)	
PPE use (days/month)	11.7 ± 0.2	20.3 ± 8.1	**0.001** *
Practice of physical activity (*n* = 147)			
Yes	99 (67.3%)	66 (44.9%)	**0.001** *
No	48 (32.7%)	81 (55.1%)	
Associated symptoms (*n* = 145)			
Yes	21 (14.5%)	66 (45.5%)	**0.001** *
No	124 (85.5%)	79 (54.5%)	

Abbreviation: PPE, personal protective equipment.

* Significance value p < 0.05.

The prevalence of craniocervical and otological symptoms before the pandemic, worsening of pre‐existing symptoms, and the onset during the pandemic are described in Table 3. Headache was the most prevalent symptom before the pandemic (32%). During the pandemic, otological symptoms were the most frequent for onset (54.4%). Meanwhile, orofacial pain and headache had significant worsening of pre‐existing symptoms (60.8% and 59.5%).

**TABLE 3:** Prevalence of craniocervical and otological symptoms before and during the pandemic and worsening of pre‐existing symptoms in healthcare workers during COVID‐19 (*n* = 147).

Symptom	Before *n* (%)	During (onset) *n* (%)	Worsening *n* (%)^a^	Rate of increase %
Orofacial pain	23 (15.6%)	54 (36.7%)	14 (60.8%)	134.7%
Headache	47 (32%)	65 (44.2%)	28 (59.5%)	38.2%
Neck pain	31 (21.1%)	56 (38.0%)	12 (38.7%)	80.6%
All otological symptoms	34 (23.1%)	80 (54.4%)	10 (29.4%)	135.2%
Total	135	255 (216.1%)	64 (73%)	116.1%
Otological symptoms				
Tinnitus	11 (7.5%)	17 (11.6%)	2 (18.1%)	54.5%
Ear fullness	7 (4.8%)	23 (15.6%)	2 (28.5%)	228.5%
Hearing loss	3 (2%)	8 (5.4%)	1 (33.3%)	166.6%
Dizziness	5 (3.4%)	13 (8.8%)	2 (40%)	160%
Vertigo	8 (5.4%)	19 (12.9%)	3 (37.5%)	137.5%

^a^ The frequency of worsening cases was considered based on the number of individuals reporting any of the symptoms prior to the pandemic (pre‐existing symptoms).

An increase in the intensity of orofacial pain (*p* = 0.013) and headache (*p* = 0.001) was observed during the pandemic. Additionally, the intensity of otological symptoms increased during the pandemic for dizziness (*p* = 0.034) and vertigo (*p* = 0.0001). An increase in the average number of days per month reporting craniocervical symptoms by healthcare workers was observed during the pandemic period (Table 4).

**TABLE 4:** Average intensity of symptoms and number of days per month with the presence of craniocervical and otological symptoms before and during the pandemic in healthcare workers during COVID‐19 (*n* = 147).

Intensity of symptoms (NRS 0–10)			
Symptoms	Before pandemic (Mean ± SD)	During pandemic (Mean ± SD)	*p*
Orofacial pain	4.6 ± 1.5	6.0 ± 2.2	**0.013** *
Headache	4.9 ± 1.9	6.2 ± 2.1	**0.001** *
Neck pain	5.0 ± 1.4	5.6 ± 1.6	0.077
Otological symptoms			
Tinnitus	3.7 ± 1.9	4.0 ± 1.9	0.510
Ear fullness	4.3 ± 3.2	4.7 ± 2.7	0.160
Hearing loss	5.0 ± 3.0	4.9 ± 2.6	0.860
Dizziness	4.2 ± 0.8	5.7 ± 2.1	**0.034** *
Vertigo	4.8 ± 1.8	4.5 ± 2.7	**0.001** *
Average number (days/month)			
Orofacial pain	7.8 ± 5.3	12.2 ± 7.8	**0.010** *
Headache	5.8 ± 4.0	11.7 ± 7.5	**0.001** *
Neck pain	8.9 ± 7.6	15.0 ± 8.2	**0.002** *
Otological symptoms	6.3 ± 5.2	8.2 ± 7.3	0.150

Abbreviations: NRS, numerical rating scale; SD, standard deviation.

* Significance value *p* < 0.05.

The onset of symptoms during the pandemic and sick leave due to COVID‐19 was not associated with orofacial pain (*p* = 0.24), neck pain (*p* = 0.17), and otological symptoms (*p* = 0.15). Similarly, the worsening of pre‐existing symptoms was not associated with sick leave due to COVID‐19 for orofacial pain (*p* = 0.44), neck pain (*p* = 0.19), and otological symptoms (*p* = 0.52).

Table 5 presents the results of the binary logistic regression model adjusted for age and sex. Considering the onset of symptoms, the presence of neck pain (OR = 2.42, 95% CI 1.03–5.71) and otological symptoms during the pandemic (OR = 3.14, 95% CI 1.29–7.65) were associated factors for orofacial pain. As for headache, only the presence of depressive symptoms (OR = 3.15, 95% CI 1.67–9.53) was noted. For neck pain, associated symptoms (OR = 3.99, 95% CI 1.67–9.53) and orofacial pain during the pandemic (OR = 2.45, 95% CI 1.06–5.61) were considered. For otological symptoms, the use of a surgical mask before the pandemic for over 4 h a day (OR = 3.06, 95% CI 1.18–7.93), use of a face shield during the pandemic for over 4 h a day (OR = 2.98, 95% CI 1.07–8.24), and the presence of orofacial pain during the pandemic (OR = 3.89, 95% CI 1.48–10.19) were identified.

**TABLE 5:** Binary logistic regression analysis of factors associated with the onset of new symptoms in healthcare workers during COVID‐19.

Variables	OR (95% CI)	*p*
Orofacial pain		
Otological symptoms during pandemic		
No	1	
Yes	3.14 (1.29–7.65)	0.011
Neck pain during pandemic		
No	1	
Yes	2.42 (1.03–5.71)	0.042
Headache		
Depressive symptoms		
Unlikely	1	
Probable	3.15 (1.05–9.39)	0.039
Neck pain		
Associated symptoms		
No	1	0.002
Yes	3.99 (1.67–9.53)	
Orofacial pain during pandemic		
No	1	0.034
Yes	2.45 (1.06–5.61)	
Otological symptoms		
Use of surgical masks before pandemic (hours/day)		
Up to 4 h/day	1	
More than 4 h/day	3.06 (1.18–7.93)	0.021
Use of face shield during pandemic (hours/day)		
Up to 4 h/day	1	0.035
More than 4 h/day	2.98 (1.07–8.24)	
Orofacial pain during pandemic		
No	1	
Yes	3.89 (1.48–10.19)	0.006

Abbreviations: CI, confidence interval; OR, odds ratio.

Table 6 reports the results of the binary logistic regression adjusted for age and sex with factors potentially associated with the worsening of pre‐existing symptoms. For orofacial pain an odds ratio of 4.72 was reported for working in the nursing ward (95% CI 1.31–16.92). Female sex (OR = 4.23, 95% CI 1.01–18.98), lack of physical activity (OR = 3.25, 95% CI 1.07–9.95) and previous medical history (OR = 3.74, 95% CI 1.14–12.26) were potentially associated with the worsening of headache. Worsening of neck pain and otological symptoms showed no associated factors.

**TABLE 6:** Binary logistic regression analysis of factors associated with worsening of pre‐existing symptoms in healthcare workers during COVID‐19.

Variables	Odds ratio (95% CI)	*p*
Orofacial pain		
Nursing ward		
No	1	
Yes	4.72 (1.31–16.92)	0.017
Headache		
Sex		
Male	1	
Female	4.23 (1.01–18.98)	0.048
Physical activity during pandemic		
Yes	1	
No	3.25 (1.07–9.95)	0.038
Previous medical history		
No	1	
Yes	3.74 (1.14–12.26)	0.023

## 4 Discussion

This study aimed to investigate factors associated with craniocervical and otological symptoms among healthcare workers during the COVID‐19 pandemic. The findings revealed a high prevalence of otological symptoms and headache, in addition to a significant increase in the intensity of orofacial pain, headache, dizziness and vertigo. Depressive symptoms, associated symptoms (nausea, vomiting and low back pain), and co‐occurrence of craniocervical and otological symptoms during the pandemic were associated with the onset of craniocervical symptoms. The use and duration of surgical masks before the pandemic and face shield and orofacial pain during the pandemic were associated factors for the onset of otological symptoms. Working in the nursing ward, female sex, lack of physical activity and previous medical history were associated with the worsening of pre‐existing craniocervical symptoms.

### 4.1 Headache and Biopsychosocial Factors

Consistent with previous studies, a high prevalence of headache among healthcare workers during the pandemic was observed [12, 26, 27, 28]. Notably, pre‐existing headache worsened in more than half of healthcare workers, with new cases emerging during the pandemic, corroborating the literature [14, 29, 30, 31]. Mechanical compression over craniofacial and cervical structures, combined with occupational stress, appears to be a relevant mechanism for the development or aggravation of headaches [12, 32, 33]. Our regression analysis highlights female sex, lack of physical activity and previous medical history as significant contributors, aligning with evidence that headache prevalence, particularly migraine, is higher in women and exacerbated by stress [34, 35]. Reduced physical activity during the pandemic, driven by social distancing, lockdowns and the closure of exercise facilities, likely contributed to increased symptom frequency and intensity. Furthermore, although the risk of headache was reported higher with longer duration of PPE use [26]; our analysis did not corroborate this finding.

### 4.2 Orofacial Pain and TMD

In our study, 59% of participants reported worsening of pre‐existing orofacial pain, and 36% experienced onset during the pandemic. Previous studies reported increases in TMD symptoms, TMJ pain, masticatory muscle pain and muscle tension [36, 37]. Particularly among healthcare workers working in wards, where prolonged exposure to stress and emotional overload can exacerbate or trigger TMD symptoms, negatively impacting psycho‐emotional health [36, 37, 38]. In our study, working in nursing wards appeared to contribute to the worsening of orofacial symptoms. Additionally, the onset of orofacial pain was associated with neck pain and otological symptoms, aligning with evidence linking these conditions [39, 40, 41].

### 4.3 Neck Pain

Approximately 38% of participants reported worsening of pre‐existing neck pain and a similar proportion experienced onset. Several studies report that neck pain in healthcare workers, regardless of a pandemic context, arises from demographic, ergonomic, psychosocial and organisational factors [42, 43]. In a pandemic context, previous studies [14, 30] also reported high rates of neck pain, including 73% in general healthcare workers, 66.7% in frontline workers and 35% in podiatrists, with contributing factors such as increased workload, poor ergonomics, irregular shifts, inadequate rest and psychosocial stress. Cervical spine symptoms may affect the stomatognathic system via the trigeminocervical nucleus [20], and myofascial trigger points in the upper trapezius may contribute to masticatory muscle imbalance. These findings suggest a bidirectional relationship between neck pain and TMD, reinforcing the importance of considering musculoskeletal interconnections in clinical assessment [44]. Furthermore, prolonged computer use by healthcare workers during the COVID‐19 pandemic has also been strongly linked to a higher likelihood of developing pain and dysfunction in the upper musculoskeletal regions [45].

### 4.4 Otological Symptoms

Our data highlight the prevalence of otological symptoms during the pandemic in healthcare workers, including tinnitus, ear fullness, hearing loss and vertigo. In addition, 29.4% of pre‐existing otological symptoms worsened, and a 54.4% incidence was observed during the pandemic. The co‐occurrence of otological symptoms with headache and orofacial pain further emphasises the interconnected nature of craniocervical and vestibular manifestations [41, 46]. The use of face masks for more than 4 h per day before the pandemic and the use of face shields for more than 4 h per day during the pandemic were associated with the development of otological symptoms. Evidence suggests that the onset of these symptoms may also be associated with prolonged use of PPE through worsened middle‐ear pressures and Eustachian tube dysfunction [47]. Moreover, stress and workload may have contributed to these complaints, possibly through mechanisms involving the trigeminocervical system and the interplay between vestibular and temporomandibular symptoms [48]. Current evidence also reports dizziness in a substantial proportion of healthcare workers using N95 masks [49].

### 4.5 Clinical Implications

Our findings highlight the need for strategies that promote both physical and mental health among healthcare workers, especially in high‐demand situations such as a pandemic. Interventions may include encouragement of regular physical activity, psychological support programs, structured breaks during shifts, ergonomic optimisation and protocols for safe and comfortable PPE use. Addressing biopsychosocial factors may mitigate the burden of craniocervical and otological symptoms and improve the quality of care.

### 4.6 Strengths and Limitations

Some limitations should be acknowledged. The cross‐sectional design does not allow causal inferences, and recall bias may have arisen from retrospective questions referring to the period before the pandemic. The relatively small sample, predominantly from the Northeast region, may not fully represent the Brazilian healthcare workforce, limiting external validity. Finally, some variables were excluded from the regression models due to small cell counts, which may have restricted model adjustment. Nonetheless, this study included a more comprehensive investigation of craniocervical and otological symptoms, using validated instruments, a nationwide sample, and multivariate analyses adjusted for age and sex.

## 5 Conclusion

During the COVID‐19 pandemic, healthcare workers experienced both worsening of pre‐existing symptoms and onset of craniocervical and otological complaints. Factors associated with the onset of craniocervical symptoms were linked to co‐occurring symptoms, depressive symptoms and associated symptoms. Meanwhile, the worsening of pre‐existing craniocervical symptoms included work department, female sex, lack of physical activity. The onset of otological symptoms was associated with prior use and duration of surgical mask use, face shield use during the pandemic and concurrent orofacial pain. Worsening of neck pain and otological symptoms showed no associated factors. High rates of craniocervical and otological complaints may negatively affect healthcare workers' quality of life, potentially impacting the care provided in public and private health institutions. Longitudinal studies with larger samples are needed to confirm these findings and to guide effective prevention and management strategies to reduce the healthcare burden.

## Author Contributions

Conceptualisation: Anita Almeida Gonzaga, Jade Louise Alves Macedo Padilha Silva, Karyna Myrelly Oliveira Bezerra Figueiredo‐Ribeiro; Methodology: Anita Almeida Gonzaga, Jade Louise Alves Macedo Padilha Silva, Rafaella Silva dos Santos Aguiar Gonçalves, Karyna Myrelly Oliveira Bezerra Figueiredo‐Ribeiro; Data collection: Anita Almeida Gonzaga, Jade Louise Alves Macedo Padilha Silva, Karyna Myrelly Oliveira Bezerra Figueiredo‐Ribeiro; Data analysis: Anita Almeida Gonzaga, Jade Louise Alves Macedo Padilha Silva, Rafaella Silva dos Santos Aguiar Gonçalves, Luiz Felipe Tavares, Álvaro Campos Cavalcanti Maciel, Karyna Myrelly Oliveira Bezerra Figueiredo‐Ribeiro; Writing – original draft preparation: Anita Almeida Gonzaga, Jade Louise Alves Macedo Padilha Silva, Álvaro Campos Cavalcanti Maciel, Karyna Myrelly Oliveira Bezerra Figueiredo‐Ribeiro; Writing – review and editing: Anita Almeida Gonzaga, Rafaella Silva dos Santos Aguiar Gonçalves, Luiz Felipe Tavares; Supervision: Álvaro Campos Cavalcanti Maciel, Karyna Myrelly Oliveira Bezerra Figueiredo‐Ribeiro. All authors read and approved the final manuscript. All authors agree to be accountable for all aspects of the work.

## Ethics Statement

This study was approved by the local research ethics committee of the Federal University of Rio Grande do Norte (number 36193520.9.0000.5537). All research procedures were in accordance with the resolution 466/12 of the National Health Council and the Helsinki Declaration. A written informed consent was obtained from each participant.

## Conflicts of Interest

The authors declare no conflicts of interest.

## Data Availability

The data that support the findings of this study are available on request from the corresponding author. The data are not publicly available due to privacy or ethical restrictions.
